# Supplementary material for: Vitamin D Supplementation and Adherence to World Cancer Research Fund (WCRF) Diet Recommendations for Colorectal Cancer Prevention: A Nested Prospective Cohort Study of a Phase II Randomized Trial
Source: Biomedicines. 2023 Jun 20;11(6):1766. doi: 10.3390/biomedicines11061766 (PMC10296113; doi:10.3390/biomedicines11061766)
Supplement: Supplementary file 1 [file biomedicines-11-01766-s001.zip › biomedicines-2390187-supplementary.pdf]

Supplementary Materials:

Supplementary Table S1 list of the particiopants' events

arms	Data random	Data first event	Type of event
Placebo	06/09/2016	07/05/2021	polyp
	06/10/2016	31/08/2020	polyp
	14/11/2016	13/12/2021	polyp
	13/12/2016	31/05/2022	relapse
	03/02/2017	10/01/2022	polyp
	20/02/2017	06/05/2021	other
	02/05/2017	27/10/2021	polyp
	18/09/2017	07/03/2022	polyp
	06/06/2017	17/05/2022	polyp
	07/09/2017	18/02/2021	polyp
	05/04/2018	04/06/2019	polyp
	06/03/2018	09/04/2020	other
	12/11/2018	17/01/2020	other
	08/06/2018	22/03/2019	relapse
	23/10/2018	17/09/2020	polyp
Vitamin D	01/07/2016	01/06/2021	other
	22/07/2016	18/05/2020	polyp
	29/07/2016	11/09/2020	polyp
	05/08/2016	10/02/2020	polyp
	29/09/2016	15/10/2020	polyp
	17/10/2016	30/11/2021	polyp
	03/03/2017	06/05/2021	polyp
	09/03/2017	06/05/2021	relapse
	26/06/2017	25/05/2022	polyp
	26/07/2017	05/05/2021	relapse
	19/10/2017	12/11/2019	polyp
	27/04/2018	31/07/2018	relapse
	08/06/2018	15/07/2020	polyp
	23/03/2018	29/06/2021	polyp
	01/08/2018	08/02/2019	polyp
	18/02/2019	01/11/2019	relapse

Relapse: can be either local recurrences or distant metastasis
Other cancers: 2 breast cancer 1 melanoma and 1 urothelial cancer

Supplementary Table S2 Biomarkers changes after intervention by arms

	Placebo			Vitamin D			
**Variable**	**Median**	**Lower Quartile**	**Upper Quartile**	**Median**	**Lower Quartile**	**Upper Quartile**	**P-values**
**VDBP** μg/mL	-9.0	-35.0	25.0	9.0	-13.0	46.0	0.1272
**25(OH)D** ng/mL	0.7	-2.9	4.7	18.5	10.3	25.8	<.0001
**Adiponectin** μg/mL	0.3	-1.1	1.9	0.3	-0.6	1.4	0.8464
**Leptin** ng/mL	1.0	-1.2	9.1	-0.2	-2.3	8.1	0.938
**IL_10** pg/mL	0.1	-0.3	0.7	0.1	-0.2	0.6	0.0884
**IL_6** pg/mL	0.1	-0.7	0.9	0.0	-0.4	1.6	0.3117
